# Inducing Tumor Suppressive Microenvironments through Genome Edited CD47^−/−^ Syngeneic Cell Vaccination

**DOI:** 10.1038/s41598-019-56370-6

**Published:** 2019-12-27

**Authors:** Subhadra Jayaraman Rukmini, Huanjing Bi, Puloma Sen, Benjamin Everhart, Sha Jin, Kaiming Ye

**Affiliations:** Department of Biomedical Engineering, Center of Biomanufacturing for Regenerative Medicine, Watson School of Engineering and Applied Science, Binghamton University, State University of New York (SUNY), Binghamton, NY 13902-6000 USA

**Keywords:** Cancer microenvironment, Cancer immunotherapy

## Abstract

Tumors can escape from the immune system by overexpressing CD47 and other checkpoint blockades. CD47 is expressed ubiquitously by all cells in the body, posing an obstacle for CD47 blocking treatments due to their systemic toxicity. We performed a study to determine how the tumor microenvironment changes after vaccination with genome edited CD47^−/−^ syngeneic tumor cells. We discovered that inactivated CD47-depleted mouse melanoma cells can protect mice from melanoma. Our animal study indicated that 33% of vaccinated mice remained tumor-free, and 100% of mice had 5-fold reduced growth rates. The characterization of immunomodulatory effects of the vaccine revealed a highly anti-tumorigenic and homogenous microenvironment after vaccination. We observed consistently that in the tumors that failed to respond to vaccines, there were reduced natural killer cells, elevated regulatory T cells, M2-type macrophages, and high PD-L1 expression in these cells. These observations suggested that the tumor microenvironments became more suppressive to tumor growth after vaccination, suggesting a potential new immunotherapy for solid tumors.

## Introduction

Cancer cells possess a plethora of immune evasion mechanisms in reaction to specific immune responses^1,2^. However, the inherent heterogeneity of solid tumor microenvironment (TME) has posed significant obstacles to the development of efficient immunotherapies^3,4^. This heterogeneity is contributed by alterations in genomic, proteomic, and epigenetic profiles^5–7^ of the TME components, including the tumor cells themselves, the fibroblasts^8–10^ and adipocytes^11,12^ surrounding the tumor, remodeling of the extracellular matrix^9^, neovasculature surrounding the tumor^13,14^, and the immune cells infiltrating the tumors^15–18^. Most adaptive changes in the tumor are attributed to the immune infiltrates. This heterogeneity petitions the need for personalized treatment measures for solid tumors – an approach that can target interpatient variability^19^.

Vaccination is one of the most explored methods for increasing immune infiltrates into solid tumors^20^. Some vaccine formulations studied previously are mRNA mutanomes^19^, tumor-associated neoantigen peptide cocktails^21^, tumor cell lysates containing immune system stimulants^22,23^, or even whole-cells^24,25^, all of which rely on extensive genotyping of individual tumors to gain knowledge of tumor-specific antigens, or extraction and modification of sensitive immune cells. Most of these mutanome-based vaccines target the cancer immunity cycle at the T cell infiltration and activation stage^19^.

We hypothesized that (i) providing the immune system with non-replicating tumor cells would circumvent the need for tumor-associated antigen (TAA) profiling, protein purification, viral packaging, and other preparation regimes^26,27^; and (ii) targeting the cycle upstream of T cell activation would lead to a lasting response that can be utilized in subsequent therapies.

To design a system to test the hypotheses, we chose one of the most studied immune checkpoint mechanisms in cancer - the CD47-SIRPα interaction^28–30^. CD47, a ubiquitous cell-surface antigen, is reported to act as a marker of self and by corollary, a “don’t eat me” signal. It interacts with the signal recognition protein alpha (SIRP-α), present primarily on macrophages^29^. This interaction halts macrophage-mediated phagocytosis and blocks any further immune responses. Tumor cells overexpress CD47 on their surface as a defense mechanism to blindside the host’s immune defense systems^31,32^. CD47 has been targeted primarily for developing checkpoint blocking antibody therapy. For instance, the blocking of CD47-SIRPα interaction using anti-CD47 monoclonal antibodies^33–36^, anti-SIRPα antibodies^33,37^, or nanobodies^28,35,38^, has shown delayed tumor progression. Various studies have shown that the depletion of CD47 expression on cancer cells using either siRNA^31,32^ or genetic editing has proven effective in slowing down tumor growth and enhancing phagocytosis by macrophages^34,37^. These studies have been essential understanding the role of CD47 in tumor immunology, and have yielded a number of formulations for combinatorial approaches^33,34,36,38,39^. However, these studies have not comprehensively elucidated immune response mechanisms and have not been able to provide an appropriate solution to the toxicity problems posed by anti-CD47 antibodies in the clinical setting^40–42^.

In this study, we reconfigured the use of CD47 as an immunotherapeutic target for developing a new treatment. When a previously dormant immune system incapable of recognizing and attacking a growing tumor is presented with syngeneic inactivated tumor cells lacking cell-surface CD47, it will lead to the mobilization of circulating macrophages. These macrophages are now capable of recognizing and phagocytosing the CD47-null tumor cells and presenting antigens effectively to T cells, leading to the development of a robust downstream immune response. This will further lead to increased infiltration of primed tumor-specific cytotoxic T cells into the microenvironment of the growing tumor, resulting in tumor shrinkage or blockade of tumor growth. Despite the presence of CD47 in the growing tumor, the immune response that now consists of tumor-specific cytotoxic T cells, will prove to be efficient in curbing further growth. This process is depicted in Supplementary Fig. 1.

We developed CD47^−/−^ mouse melanoma B16F10 cell lines through CRISPR/Cas9 gene editing and inactivated them using gamma irradiation for vaccination. We hypothesized that these CD47 edited vaccines induce a tumor suppressive environment to suppress or reduce the tumor progression.

## Results

### Validation of CD47 as a target for vaccine development

**Figure 1:**
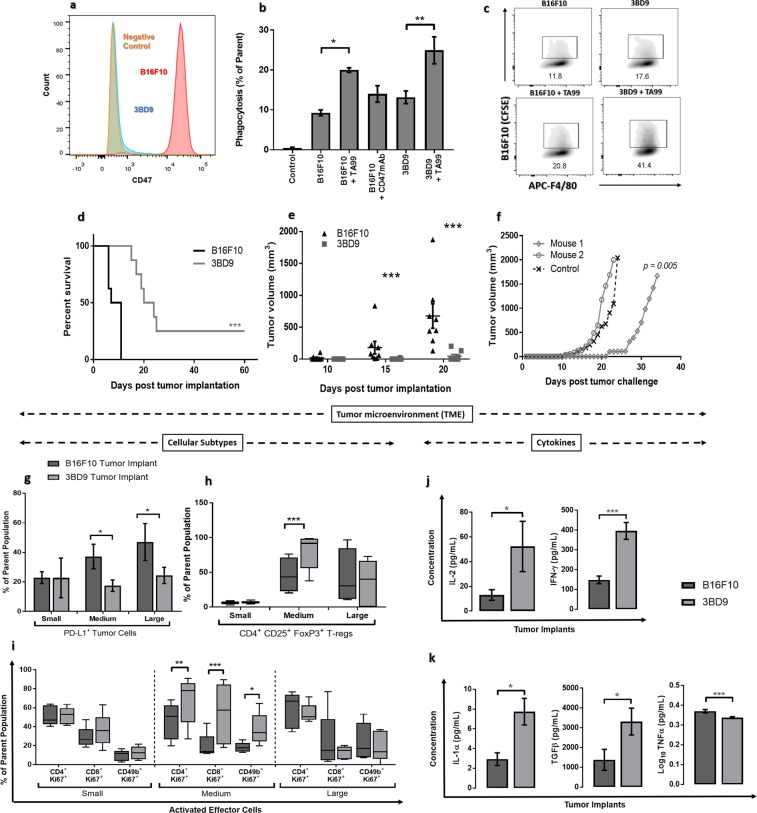
Validation of CD47 as a target for vaccine development. (**a**) Flow cytometry histograms showing the CD47 expression in B16F10 cells (red – positive control), 3BD9 cells (blue), and a negative control (orange). (**b**) Comparison of phagocytosis of B16F10 cells and 3BD9 cells in the presence and absence of the opsonizing antibody, TA99. The data shown are the mean (n = 3) and the error bars indicate the standard error. *p < *0.05*, **p < *0.01*, one-way ANOVA. (**c**) Representative flow cytometric density plots showing number of double positive (APC-F4/80^+^CFSE^+^) macrophages, depicting the percentage of phagocytosis in each condition. (**d**) Survival rate of B16F10 or 3BD9 implanted mice **p < *0.01*, (**e**) Tumor growth in B16F10 or 3BD9 implanted mice. Points indicate tumor measurements from individual mice (n = 8). ***p < *0.001*, unpaired *t* test. Error bars indicate standard error. Mantel-Cox test. (**f**) Tumor growth rate after challenge (second tumor implantation with live B16F10 cells) for two mice that were tumor-free for 60 days after initial 3BD9 implantation. *p* = *0.005* by linear regression analysis. (**g**) PD-L1 expression on tumor cells, (**h**) infiltration of regulatory T cells (T-regs), and (**i**) activated (Ki67^+^) effector cells (CD4^+^ T cells, CD8^+^ T cells, and NK cells) in the tumor microenvironment. n = 15 mice per group. Concentration profiles of cytokines (**j**) IL-2 and IFN-γ; and (**k**) IL-1α, TGFβ, and TNFα in the TME of CD47^+/+^ B16F10 and CD47^−/−^ 3BD9 tumors. n = 15 for IFN-γ and n = 3 for other cytokines. *p < *0.05*, **p < *0.01*, ***p < *0.001* by one-way ANOVA using GraphPad Prism. Flow cytometric analysis was performed using FlowJo.

We knocked out the *cd47* gene from the B16F10 cell line using a dual-guide gene deletion protocol by CRISPR/Cas9 genome editing. Edited cells were screened for bi-allelic CD47 knockout by PCR and DNA sequencing (Supplementary Table 1) and were quantified through flow cytometry (Fig. 1a). The resultant single cell clone was named as 3BD9 that was used in the subsequent experiments. We performed an *in vitro* phagocytosis assay to determine engulfment of 3DB9 cells by bone marrow derived-macrophages (BMDMs) in the presence of an opsonizing antibody TA99 (anti-gp75, a common melanoma tumor-associated antigen)^36^. The phagocytosis was enhanced considerably in the presence of TA99 (Fig. 1b,c), suggesting the combinatory effect of CD47 absence and antibody opsonization.

We next examined tumor growth by implanting CD47^−/−^ 3BD9 cells in syngeneic immunocompetent C57BL/6 mice^34^. Two of the eight mice (25% of mice) implanted with 3BD9 cells did not develop a tumor up to 60 days post implantation (Fig. 1d). In the mice that developed tumors, growth was delayed by at least 10 days in comparison with the mice implanted with CD47^+/+^ B16F10. (Fig. 1e). To determine whether these tumor-free mice developed an immune memory against melanoma, we performed a second tumor implantation with CD47^+/+^ B16F10 cells on Day 61. Interestingly, one mouse showed significantly delayed tumor growth - by about 20 days (Fig. 1f). These experiments unveiled the possible elicitation of immune memory by CD47^−/−^ tumor cells.

To characterize the immune activity in CD47^−/−^ tumors, we used an additional cohort of 15 mice per group that received B16F10 implants and 3BD9 implants subcutaneously. We performed immunophenotyping to characterize different immune cell subsets in the TME and in the tumor-draining lymph nodes (TDLNs) of mice (Supplementary Table 2) using cell-specific markers (Supplementary Table 3). This revealed a significant increase in tumor cell surface PD-L1 expression as tumors progressed in B16F10 engrafted mice - from 20% at early stage to 45% at final stage - suggesting the gradual development of an immunosuppressive environment (Fig. 1g). In contrast, PD-L1 expression in CD47^−/−^ 3BD9 engrafted mice remained steadily low as tumors grew. CD47^−/−^ tumors also exhibited a higher level of regulatory T cell (T-reg) (Fig. 1h), Ki67^+^ proliferating T cell and natural killer (NK) cell (Fig. 1i) infiltration when the tumors grew to a size of 500–600 mm^3^, suggesting that there is a phase of tumor growth when the host immune system responds to the CD47^+/+^ and CD47^−/−^ tumors differently. Correspondingly, the cytokine profiles of the CD47^+/+^ and CD47^−/−^ tumors were significantly different. In the CD47^−/−^ 3BD9 tumor microenvironment, there was a substantial increase in IL-2 and IFN-γ, the cytokines primarily associated with T cell health and deemed indispensable for T-reg cell activity and induction (Fig. 1j). Furthermore, IL-1α, which orchestrates the conversion of FoxP3 CD4T cells to FoxP3^+^ T-reg and TGF-β, which is known to be the primary regulator of T-reg induction and function were both elevated in the CD47^−/−^ 3BD9 tumors (Fig. 1k). Another important cytokine, TNFα, which is known to impair TGF-induced T-reg function was found to reduce in the tumor microenvironment of CD47^−/−^ 3BD9 tumors (Fig. 1k). These immunomodulatory cytokines are also responsible for an increase in the T cell subsets, orchestrating phenotypic shifts in the CD4 T cell subtypes, and play a role in the increasing cytotoxic T cell activity in the tumor microenvironment of CD47 deficient tumors.

### Vaccination with inactivated CD47^−/−^ tumor cells induces a tumor suppressive microenvironment that suppresses tumor growth and reduces tumor progression

**Figure 2:**
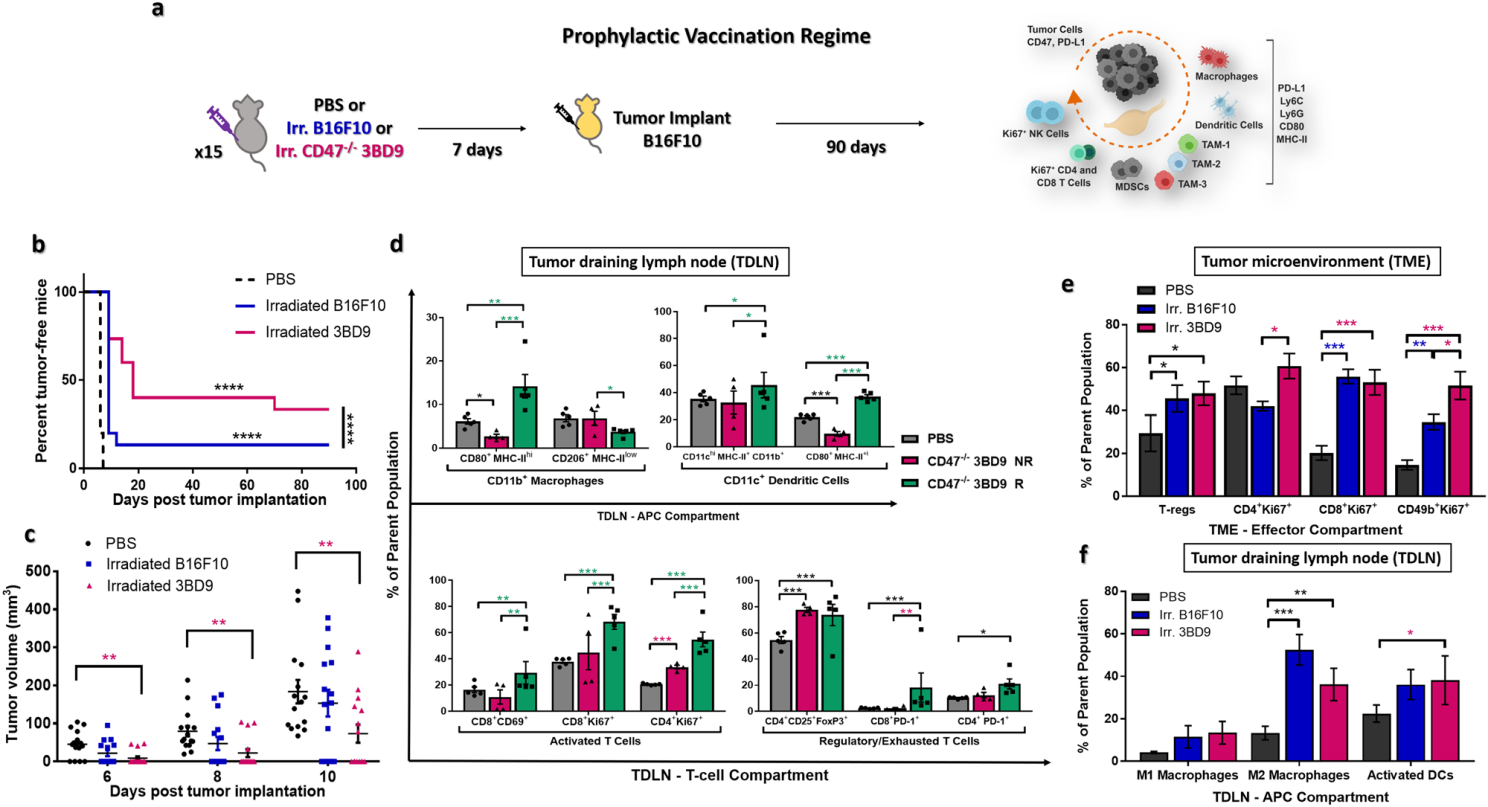
Prophylactic vaccination with inactivated CD47^−/−^ syngeneic tumor cells. (**a**) The study regime: 15 mice per group were vaccinated with PBS, irradiated CD47^+/+^ B16F10, or irradiated CD47^−/−^ 3BD9 cells, and challenged with live B16F10 cells 7 days later. Mice were observed for 90 days post tumor implantation. Tumors and lymph nodes were collected and immunophenotyping was performed to analyze various lymphocyte populations. (**b**) Percentage of tumor free mice after vaccination. ****p < *0.0001* by the Mantel-Cox test. (**c**) Tumor growth in mice from the three vaccination regimes. **p < *0.01* by unpaired *t* test. (**d**) Macrophage and dendritic cell (DC) subsets present in the antigen presenting cell (APC) compartment, CD8^+^ and CD4^+^ T cell subsets in the T-cell compartment of TDLNs. Symbol: *NR*, *non-responders (mice that grew tumors after vaccination); R*, *responders (tumor-free mice after vaccination). n* = 5 *mice per cohort for PBS and R groups. n* = 4 for NR group. *p < *0.5*, **p < *0.01*, ***p < *0.001 by the unpaired t test performed using GraphPad Prism*. (**e**) Regulatory T cells (T-regs), proliferating (Ki67^+^) T cells and NK cells in the tumor microenvironment, and (**f**) macrophage (M1- and M2-type) and dendritic cell subsets in the tumor-draining lymph nodes of mice in the PBS, 3BD9, and B16F10 vaccinated groups. For (**e**) and (**f**) - *In the PBS cohort*, *n* = *15. In the 3BD9 cohort*, *n* = 9 *and in the B16F10 cohort n* = 13. *p < *0.05*, **p < *0.01*, ***p < *0.001 by unpaired t test performed on GraphPad Prism*. Flow cytometric analysis was performed on FlowJo and cell phenotypes are presented as a percentage of the parent cell population.

Upon confirming the immune response to CD47^−/−^ cancer cells, we next sought to determine whether γ-irradiate, non-replicating CD47^−/−^ cancer cells can be used as a vaccine to suppress tumor growth and reduce tumor progression. As controls, mice vaccinated with PBS and irradiated B16F10 (CD47^+/+^) cells were studied alongside. Mice were vaccinated subcutaneously with irradiated 3BD9 or B16F10 cells, on their left flanks and challenged with live B16F10 cells on the same flank 7 days later (Fig. 2a). In the 3BD9 vaccinated group, 40% of the mice (6/15) were tumor-free until day 70 post tumor challenge, and 33% of the mice (5/15) were tumor-free until the end of the regime (90 days post tumor challenge), whereas only 13% of mice (2/15) vaccinated with B16F10 cells were tumor free until the end of the regime (Fig. 2b). The tumors that did develop after 3BD9 vaccination, showed significant delay in growth – 1.8-fold reduced tumor sizes (Fig. 2c). These data suggested a strong tumor-specific immune response due to CD47^−/−^ tumor cell vaccination. It warranted a further study to unlock the underlying mechanisms.

To this end, we compared the immune cell populations in the TDLNs of the mice that were tumor-free - responders (CD47^−/−^ 3BD9 R), and those that developed tumors after vaccination - non-responders (CD47^−/−^ 3BD9 NR) (Supplementary Table 2). We observed a four-fold increase in the M1-type anti-tumorigenic macrophages and a two-fold reduction in the M2-type pro-tumorigenic macrophages in the responders when compared with the non-responders. Additionally, activated dendritic cells (DCs) were six times higher in the responders (Fig. 2d), suggesting efficient antigen presentation in their TDLNs. Activated T cells were also elevated in the responders (Fig. 2d), confirming the presence of a long-term highly anti-tumorigenic immune response. It is, however, interesting to note that the T-reg and the exhausted T cell (PD-1^+^) populations were higher in the responders. Targeting the first responders while vaccinating leads to an efficient and lasting downstream immune response, and these mechanistic analyses open avenues for further therapeutic intervention.

### Absence of CD47 in whole-cell vaccines promotes expansion of anti-tumorigenic immune phenotypes as tumors progress

Next, we tracked the alteration in immune phenotypes in the TME and TDLNs (Supplementary Table 2 and 3) of mice that developed tumors after vaccination. We found that the 3BD9 vaccinated mice had significantly higher percentages of Ki67^+^ T and NK cells, and T-regs. Of special interest is the consistently elevated levels of proliferating NK cells, which are one of the most impactful effector cells, and have previously been found to contribute to the macrophage pathway^43,44^. In contrast, the B16F10 vaccinated mice had much lower NK cells and CD4^+^ T cells that were proliferating (Fig. 2e). We hypothesized that the abundance in T cell populations and corresponding cytokine release, as observed in the CD47^−/−^ tumors (Fig. 1j,k), could be driving the phenotypic change to a regulatory form^45,46^. In the TDLNs of 3BD9 vaccinated mice, we detected a marginal increase in the levels of M1-type macrophages but also a significant elevation in the M2-type macrophages. The irradiated 3BD9 vaccinated mice also showed much higher levels of activated DCs (Fig. 2f). These data correspond to the overall observation that the TME is “hot” and active in the 3BD9 vaccinated tumors, and the immune activity in the TDLNs correlates with TME alterations. Some suppressor cells like T-regs and M2-macrophages were elevated at certain stages of growth, and we hypothesized that this phenotypic shift contributed to original tumor escape after vaccination.

**Figure 3:**
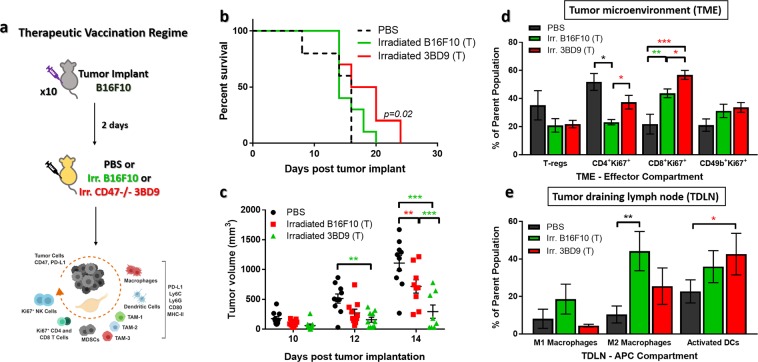
Therapeutic vaccination with inactivated CD47^−/−^ cells. (**a**) The study regime: 10 mice per group were implanted subcutaneously with B16F10 cells and treated with PBS, irradiated B16F10, or irradiated CD47^−/−^ 3BD9 cells 2 days later. Tumors and lymph nodes were collected and immunophenotyping was performed to analyze various lymphocyte populations. (**b**) Survival of mice after therapeutic vaccination. *p* = *0.02* by Mantel-Cox test. (**c**) Tumor growth in mice from the three therapeutic vaccination regimes. **p < *0.01*, ***p < *0.001* by unpaired *t* test. (**d**) Regulatory T cells (T-regs), proliferating (Ki67^+^) T cells, and NK cells in the tumor microenvironment, and (**e**) Macrophage (M1- and M2-type) and dendritic cell subsets in the tumor-draining lymph nodes of mice in the PBS, 3BD9, and B16F10 therapeutic vaccinated groups. *In all cohorts n* = 10. *p < *0.05*, **p < *0.01*, ***p < *0.001 by unpaired t test performed on GraphPad Prism*. Flow cytometric analysis was performed on FlowJo and cell phenotypes are presented as a percentage of the parent cell population.

Furthermore, we determined the suppression of tumor progression by treating tumor mice with CD47^−/−^ vaccines. We treated tumor mice with the irradiated cells (B16F10 or 3BD9) or PBS 2 days after B16F10 tumor implants. We observed tumor growth and performed immunophenotyping (Supplementary Table 2) of the TME and TDLNs (Fig. 3a) in these mice. The survival rate of mice treated with irradiated CD47^−/−^ 3BD9 cells improved marginally – 10-day increase in survival (Fig. 3b), and there was a significant delay in tumor growth – 1.5-fold reduced tumor sizes (Fig. 3c), similar to the trend observed in the prophylactic vaccination regime. To understand the underlying mechanisms after therapeutic vaccination, we characterized the immune infiltrates in the TME and the dominant phenotypes in the TDLN using the same panels as used for the prophylactic vaccination (Supplementary Table 3). We observed similar trends in the effector cells in the TME, with elevated levels of Ki67^+^ proliferating T cells, but did not see any significant difference in the NK cell populations this time. In contrast to the prophylactic vaccination also, we did not see an increase in the regulatory T cells, and they seemed to be marginally, though not significantly, reduced (Fig. 3d). We hypothesized that NK cell mobilization and infiltration into tumors, as well as the phenotypic shift of T cells to a regulatory subset requires a longer duration for immune priming and response, and due to the aggressive nature of the mouse melanoma tumor model, the therapeutic vaccination regime did not have enough time to activate the immune system for an anti-tumor attack. Immunophenotyping the TDLNs showed a marginal decrease in M1-tpe macrophages and increase in M2-type macrophages, with a significant increase in the activated DC subsets (Fig. 3e). These phenotypic differences also correlate with the findings in the TME. Insufficient time to generate an immune response, we hypothesize, could be one of the primary reasons why the vaccines only show a marginal improvement in survival, and additional dosage regimes and combination approaches need to be explored to understand this mechanism better.

### Suppressive TIL phenotypes are reduced and display distinct phenotypes after 3BD9 vaccination

**Figure 4:**
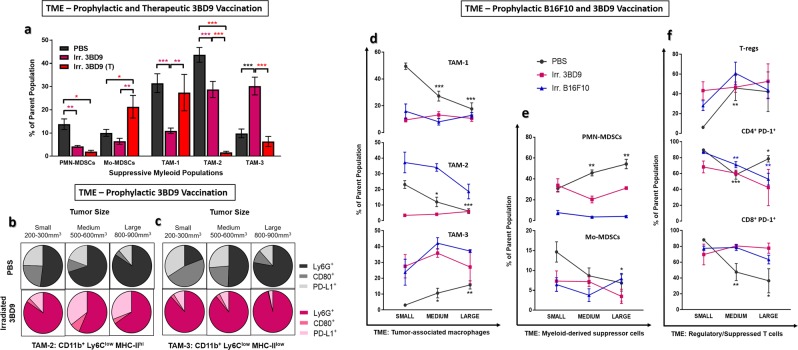
Alterations in the tumor microenvironment in 3BD9 vaccinated mice with distinct suppressor cell phenotypes. Multicolor flow cytometric analysis was performed to determine (**a**) infiltration of TAMs and MDSCs in PBS vaccinated and 3BD9 vaccinated (prophylactic and therapeutic) mice, and (**b-c**) the expression of lineage (Ly6G), activation (CD80), and T cell suppression (PD-L1) markers on the surface of MHC-II^hi^ TAM and MHC-II^low^ subsets in PBS and 3BD9 vaccinated (prophylactic) mice at three stages of tumor growth - small (200-300 mm^3^), medium (500-600 mm^3^), and large (800-900 mm^3^). Additionally, tumors at the three different stages of growth were analyzed for suppressive TIL populations in PBS vaccinated (black lines), irradiated B61F10 vaccinated (blue lines), and irradiated 3BD9 vaccinated (pink lines). (**d**) Tumor-associated macrophages (TAMs), (**e**) myeloid derived suppressor cells (MDSCs), and (**f**) T-regs (CD4^+^CD25^+^FoxP3^+^) and PD-1^+^ T-cells are shown. For (**a**), PBS n = 15; 3BD9 n = 9; and 3BD9-T n = 10. In the PBS vaccination cohort, n = 5 for all three stages of tumor. In the 3BD9 prophylactic cohort, n = 4 (small), n = 3 (medium), and n = 2 (large). In the B16F10 prophylactic cohort, n = 5 (small), n = 4 (medium), n = 4 (large). Immune cell phenotypes are presented as a percentage of the parent cell population. *p < *0.05*, **p < 0.01, ***p < 0.001 by unpaired *t* test performed on GraphPad Prism.

Next, we characterized various subsets of suppressor cells – the tumor associated macrophages (TAMs) and the myeloid derived suppressor cells (MDSCs) in the tumor microenvironment. We used a very specific gating strategy to interrogate TAMs based on expression of the granulocytic lineage marker Ly6G, the monocytic marker Ly6C, and the antigen presentation complex MHC-II (Supplementary Fig. 2). Firstly, there were distinct differences between the suppressor cell subsets in the TME of tumors treated by the prophylactic and the therapeutic regimes. It is important to note the major difference in the two regimes, which is the time given for immune cell priming and response generation. Due to this factor, and the fact that B16F10 melanoma is a very aggressive mouse tumor model, we observed significant differences in the subsets, levels, and phenotypes of immune infiltrates, especially the suppressive cells. We found that 3BD9 vaccinated tumors have overall significantly less suppressive Antigen presenting cells (APCs) in their TME. (i) In the prophylactic setting, both the polymorphonuclear (PMN) and the monocyte-derived (Mo) MDSCs are lower, whereas in the therapeutic setting, the Mo-MDSCs are elevated (Fig. 4a). (ii) In the TAM compartment, the MHC-IIhi TAMs (TAM-1 and TAM-2) are significantly lower in the prophylactic 3BD9 vaccinated tumors. The MHC-IIlow TAMs (TAM-3), however, are significantly higher (Fig. 4a). In the therapeutic setting, however, the TAM-2 and TAM-3 are significantly lower, whereas the TAM-1 is higher.

We know from various previous characterizations, that TAM-1s (Ly6C^int^) are the first TAM subtypes to be formed from monocytes, and they differentiate into TAM-2 (Ly6C^low^ MHC-II^hi^) and TAM-3 (Ly6C^low^ MHC-II^low^). TAM-3s are also usually found in hypoxic conditions, as opposed to their MHC-II^hi^ counterparts (TAM-2), which are usually present in normoxic conditions^47^. In the prophylactic vaccinated tumors, there was visible neovasculature all around the subcutaneous tumors (data not shown). Due to the difference in the time taken for this phenotypic shift, it is natural that the therapeutic and the PBS vaccinated tumors display higher levels of TAM-1 than the other subtypes, whereas the prophylactic vaccinated tumors show higher levels of TAM-2 and TAM-3. The proliferation of TAMs and their levels in the TME is also correlated with the stage of tumor growth, tumor necrosis, and neovasculature. We observed the tumors were, in general, much slower growing in the prophylactic vaccination regime than in the therapeutic (Figs. 2c and 3c), and while the presence of the precursor TAM-1s is a measure of the rapid tumor outgrowth, we also found that in the tumors that escaped after the prophylactic vaccination, eventually accrued a lot of TAM-3s, making the immune attack ineffective after a certain point in tumor growth unless other combination measures are adopted.

In the prophylactic vaccination regime, the different TAMs also showed a high phenotypic variability. With increase in tumor size, the MHC-II^hi^ TAMs in the TME of 3BD9 vaccinated tumors seemed to gradually lose Ly6G expression, produce minimal T cell stimulatory surface antigens (CD80), and elevated levels of PD-L1, in contrast to PBS vaccinated tumors (Fig. 4b). In the case of the MHC-II^low^ TAMs, the difference was more distinct. The TAMs in the 3BD9 vaccinated mice maintained a high level of Ly6G expression throughout the tumor growth phase, and negligible levels of CD80 and PD-L1. In contrast, Ly6G expression in the TAMs infiltrating tumors of the PBS vaccinated mice remained almost negligible in the early stages of tumor growth but became more significant as tumor grows (Fig. 4c). These TAMs also had increased CD80 and PD-L1 expression in comparison with vaccinated mice. These data for the inflammatory lineages of the suppressor cells were important in the quest to turn “cold” tumors “hot”.

### Vaccination increases homogeneity in TIL populations throughout tumor growth period

TIL modulation after vaccination is not limited to the difference in phenotypes of TILs. Many anti-tumor therapies rely on the percentages of TILs in tumors, and tumors often show extreme heterogeneity in infiltrating lymphocytes at various stages of development. We found that an important effect of vaccination before a tumor implant is the maintenance of stable TIL amounts throughout the development of a tumor. In particular, the percentage of suppressive phenotypes of all TILs remains constant at all stages of tumor growth in vaccinated mice, when compared to vehicle controls. Levels of TAMs and MDSCs (Fig. 4d,e), as well as T-regs and PD-1^+^ T cells (Fig. 4f) were stable in both the B16F10 and 3BD9 vaccinated tumors in comparison with the PBS vaccinated tumors. Since most immune checkpoint blockade therapies target these suppressive phenotypes, this is an important observation that could potentially alter dose escalation issues and minimize modulations in therapy.

## Discussion

CD47 has the potential to alter immune response dramatically, as it functions at one of the earliest and most crucial stages of an immune response cascade^42,48^. Additionally, the use of inactivated non-replicating tumor cells as vaccines utilizes the immunogenic potential of whole tumor cells leading us to hypothesize that cell-surface CD47 depletion can unmask whole-cell vaccines to the immune system effectively. In this preliminary proof-of-concept study, we showed the potential of inactivated CD47^−/−^ tumor cells in generating a lasting immune response.

We studied the inducing a tumor suppressive microenvironment through vaccination of genome edited CD47 tumor cells. We discovered that 33% of mice vaccinated with CD47^−/−^ melanoma cells remained tumor-free at the end of 90 days post tumor challenge. Upon extensively profiling the tumor infiltrating lymphocytes and the cells in the draining lymph nodes, we found remarkable immunomodulation orchestrated by vaccination. In the mice that did not develop tumors after vaccination, we speculated that this complete response is due to increased M1-type macrophages and activated effector T cells. However, we also discovered that there was a remarkable increase in the number of regulatory T cells as well as PD-1 expression on T cells, that led us to believe that prolonged circulation of these effector cells might cause phenotypic shifts to exhaustion. We demonstrated that the CD47 depletion results in a significant difference in the TME’s cytokine profiles, including myeloid-based cytokines such as TGFβ, IL1α, and TNFα; as well as effector cell-based cytokines IL-2 and IFNγ. Due to the increase in actively proliferating myeloid and T cell subsets, these cytokines are also elevated, except for TNFα, which was reduced, forming the perfect cocktail of factors to aid the expansion of regulatory T cells in the TME. In the future, longer study regimes, testing multiple doses and altering the number of cells per dose can help determine the most effective regime of vaccination^24,26^.

While we have an overall macroscopic view of the cellular and immuno-modulatory mechanisms orchestrated by vaccinating mice with CD47^−/−^ whole tumor cells, we have not studied the detailed mechanisms at a molecular level by way of analyzing T cell receptors (TCRs) and the specific anti-tumor cytotoxic responses in TME. The primary aims of this research were to study the alterations in the immune compartment in response to a tumor upon vaccination with CD47-depleted syngeneic cells.

We also found a significant reduction in tumor growth rates in vaccine treated tumor mice, and immune modulations similar to those observed in the prophylactic vaccinated tumors. There were a few major differences in the immune responses, however, like the phenotypic shift of T cells to a regulatory phenotype and the active involvement of natural killer cells in the anti-tumor immune response, which we believe is an indication of the time required for lasting immune responses to develop in the body. We were, overall, able to achieve a comprehensive overview of the changes that occur in the immune system when it is presented with CD47^−/−^ irradiated tumor vaccines.

A more unexpected, but very informative observation in this study was the extreme downregulation of cell surface CD47 by the tumor cells after vaccination with irradiated CD47^−/−^ cells. We found that the vaccinated tumors express almost no CD47, suggesting that the tumors might be reacting to the specific types of immune cell priming and response, which originated from cells that did not have cell surface CD47. This also suggests that the CD47 depletion from tumor cells had a very specialized effect on the anti-tumor immune response. The nuances of this can be explored in further detail by phenotyping the immune compartments more intricately and studying cytokines in the TME.

Prophylactic CD47^−/−^ whole tumor cell vaccination also caused a two-fold reduction in tumor growth rates and five-fold reduction in size in the tumors that escaped. We found that the delayed outgrowth was caused by significantly more effector cells infiltrating the tumor. The reason for tumor escape was attributed to a phenotypic shift of these T cells to more regulatory and exhausted phenotypes. Interestingly, we constantly observed an increase in tumor infiltrating NK cells in the CD47^−/−^ vaccinated mice. Presence of prominent levels of functionally activated NK cells keeps the immune response consistently anti-tumorigenic^43,44,49^. Other studies have shown the dependence of NK cell-associated cytotoxicity on CD47 expression^43,44^, and we have confirmed that the absence of CD47 leads to higher amounts of active NK cell populations, leading to tumor containment and sustained rejection.

Other principal factors in a suppressive environment are TAMs and MDSCs. They are responsible for a sizable portion of effector cell suppression in both the TME and in the TDLNs^50–53^. It has been emphasized in previous research that common characteristics of tumors that have been irreversible thus far like metastatic potential, downregulation of MHC genes, and overexpression of evasion markers, can be attributed to the specific APC phenotypes in the TME^54,55^. Prophylactic 3BD9 vaccination caused an increase in the MHC-II^low^ TAM levels, suggesting that the tumors became more hypoxic. These TAMs were also found to express high levels of Ly6G, the neutrophil lineage marker. All other suppressor cells, including the PMN- and Mo-MDSCs, were highly downregulated in the TME of CD47^−/−^ vaccinated tumors, proving that they have an overall anti-tumorigenic environment. In the therapeutic regime, we found higher levels of the precursor TAMs - the Ly6C^int^ TAM-1 subtype – further confirming our observation that in an aggressively growing tumor, the time taken for an immune response to alter plays a prominent role in the anti-tumor effects.

An important consideration in the design of therapeutic regimes for actively growing tumors is the intensity of immune activity in the tumors^56,57^. Cold tumors, hence, are often hard to treat just by therapeutics, and by corollary solid tumors are harder to treat with immunotherapeutic agents than systemic tumors. We found here that after vaccination the immune infiltration not only increases, but also maintains a consistent composition throughout the phases of tumor growth. The extreme heterogeneity that is characteristic of melanoma tumors, is reversed upon vaccination, and tumors harvested at the three stages of growth show more homogeneity in TIL populations, especially the suppressive subsets. These tumors also show a gradual increase in the effector cell compartment as tumor progresses, an effect opposite to the one seen in the tumors of mice vaccinated with inactivated B16F10 cells, which distinguishes the two types of vaccines and emphasizes the involvement of CD47 as a target in this regime. This information could help in the development of more effective therapeutic regimes.

**Figure 5:**
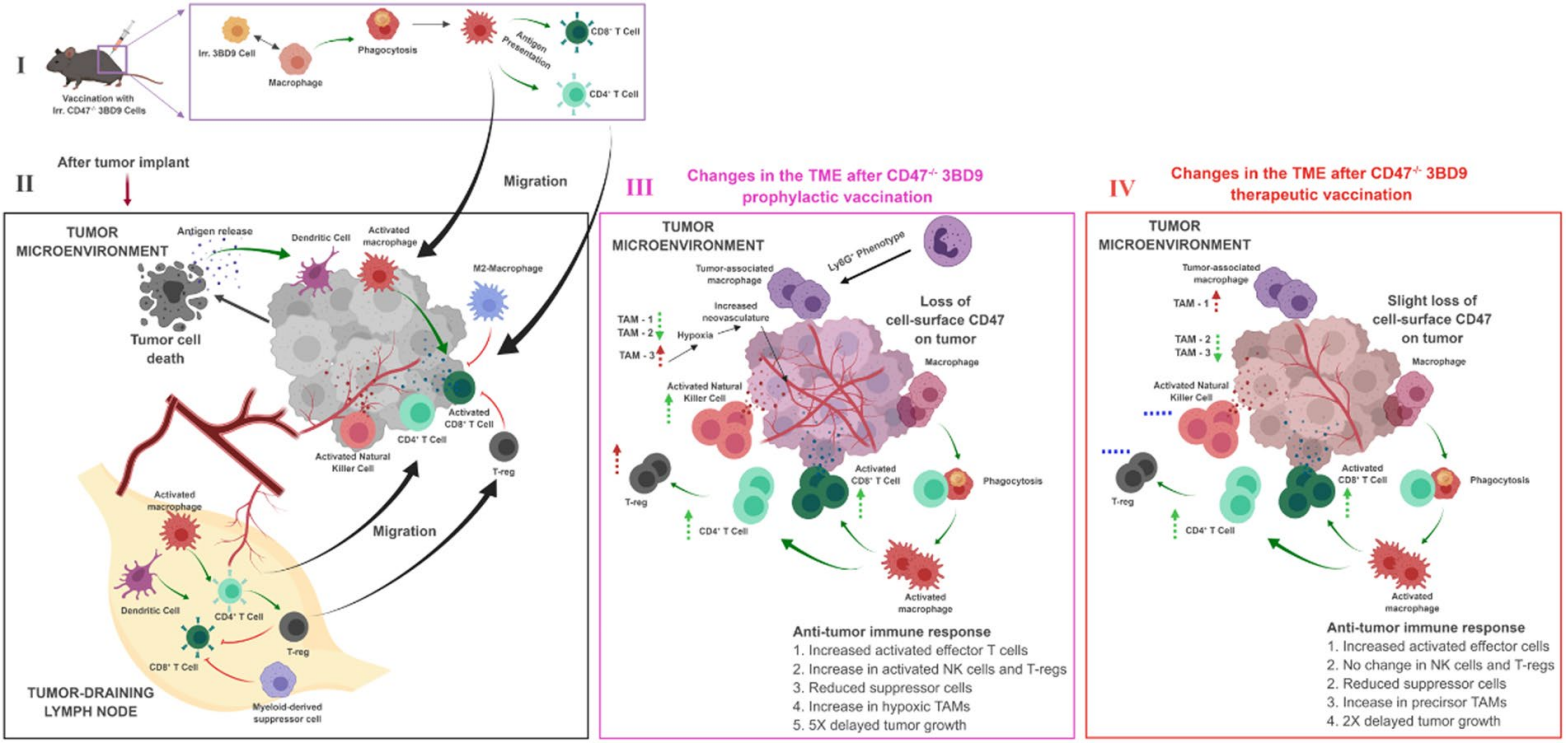
Immune response in the tumor microenvironment after prophylactic and therapeutic vaccination with 3BD9 cells. The chart depicts the immune activity as observed after vaccination with CD47^−/−^ 3BD9 cells (**I**) by analysis of the immune phenotypes infiltrating tumors typically after a tumor implant (**II**), as well as post vaccination with CD47^−/−^ 3BD9 cells, both prophylactic (**III**) and therapeutic (**IV**). Black arrows in the figure depict lineage and process, green arrows depict activation, and red lines depict inhibition. Dotted green arrows and dotted red arrows near cell subsets depict anti-tumorigenic and pro-tumorigenic cell types, respectively, and their direction depicts the levels observed (UP arrows – elevated; DOWN arrows – reduced). Blue dotted lines depict no change from control tumors (PBS vaccinated). Cell types are divided into sections based on their phenotypic characteristics. The antigen presenting cells subset shows activated M1-type macrophages and activated dendritic cells; the activated effector compartment includes Ki67^+^ T cells and NK cells; the exhausted T cell compartment consists of PD-1^+^ T cells; the tumor associated macrophage compartment includes the Ly6C^int^ TAMs (TAM-1), MHC^hi^ TAMs (TAM-2) and MHC^low^ TAMs (TAM-3).

All these outcomes proved imperative in characterizing the specifics of an anti-tumor response after vaccination with irradiated CD47^−/−^ tumor cells, which is pictorially represented in Fig. 5. We found in our studies that the right cocktail of factors that modulate cytokine release, macrophage activation, and engage tumor-specific antigens can vividly enhance the already positive effects of CD47 as an immunotherapeutic target. We can conclude that therapies targeting PD1 expressing T cells, PD-L1 expressing tumor cells and APCs, and the regulatory compartment of the T cells would be very effective in enhancing tumor rejection. We have consistently seen in this study, the involvement of the dendritic cells. The use of cytokines like GM-CSF to selectively increase the M1-type macrophages^51^ and dendritic cells and reduce the MHC^low^ TAM populations^47^ can be employed as adjunct therapies to CD47-based prophylactic vaccination regimes. The selection of the right adjuvant to enhance the specificity of the vaccines should also be explored in the future. Though the other phenotypes of exhausted T cells were not studied in this project, the expression of CTLA-4, TIM-3 and LAG-3 usually correspond to the expression of PD-1 on T cells^54,55,58^ and these can be potential combinations with the CD47 target. In the future, studying the B cell compartment^59^ and analyzing circulating antibodies and cytokines might help us understand immune memory and specific effects of the vaccines in further detail.

Here, we showed that CD47 can be employed as a vaccine target to suppress tumor growth and to increase immune infiltration in solid tumors. Further testing of efficacy in other solid tumors is necessary to establish the use of the vaccine for cancer treatment. This includes testing the vaccine on slower growing tumors that are not as aggressive as the B16F10 melanoma model, and on tumor models across the immunogenicity spectrum including colorectal carcinomas, pancreatic, prostate and breast cancer models.

## Materials and Methods

### Cell culture

The B16F10 (mouse melanoma) cell line was obtained from ATCC. The cells were cultured using high glucose DMEM (Gibco, MD) supplemented with 10% v/v Fetal Bovine Serum (FBS) (Sigma-Aldrich, MO). Mouse bone marrow derived macrophages (BMDMs) were obtained by flushing out the bone marrows of untreated mice, and culturing for 7 days in RPMI 1640 (Gibco, MD) supplemented with 10% v/v FBS.

### CD47 knockout using CRISPR

The spCas9(BB)-2A-GFP (PX458) plasmid (Addgene, MA) containing the gRNA sequence targeting CD47, and the Cas9 and an eGFP separated by a T2A sequence was transfected into the B16F10 cells using the Viafect transfection kit (Promega, WI). The successfully transfected cells were sorted as single cells into 96-well plates using a BD FACS Aria II sorter. Cells were expanded and tested for biallelic knockout of CD47 using PCR, T7E1 mismatch assay, and Sanger’s sequencing. Absence of the protein expression in edited cells was confirmed by immunofluorescence microscopy and flow cytometric analysis. Rat anti-mouse CD47 antibody clone miap301 (BD Biosciences, NJ) was used as a primary antibody to determine the absence of CD47 expression in edited cells. This antibody was used at a final concentration of 10 μg/mL (1:50 dilution). Goat anti-rat IgG tagged with AlexaFluor-488 (2 mg/mL) (Sigma-Aldrich, MO) was used as a secondary antibody. This antibody was used at a final concentration of 10 μg/mL (1:200 dilution).

### *In vitro* phagocytosis assay

Macrophages were extracted from bone marrows flushed out from the femurs of naïve mice, plated on 10 cm dishes. The growth medium was supplemented with 10 µg/mL GM-CSF. The cells were cultured for 7 days - the growth medium was replaced every 3 days. 5 × 10^4^ macrophages were co-cultured with 1 × 10^5^ carboxyfluorescein (CFSE)-labeled B16F10 tumor cells in RPMI 1640 (Gibco, MD) for 2 h at 37 °C and 5% CO_2_ in the presence of 0.5 µg of opsonizing antibody, anti-gp75 TA99 (BioXCell, NH). Macrophages were then stained with APC-tagged F4/80 (Biolegend, CA). Phagocytosis analysis was performed using a BD FACS Aria II flow cytometer.

### Tumor implants and analyses

5 × 10^5^ B16F10 (CD47^+/+^) and 3BD9 (CD47^−/−^) tumor cells were implanted subcutaneously into the left flanks of 7-week-old female C57BL/6 mice. Tumor growth was observed every alternate day and tumors were measured using a Vernier caliper. Two separate experiments with four mice/group were performed to compare the tumor growth pattern. Another cohort of fifteen mice per group was used to study tumor infiltrating lymphocytes and tumor-draining lymph node (TDLN) immunophenotypes at three different stages of tumor growth - small (200–300 mm^3^ tumors), medium (500–600 mm^3^ tumors), and large (800–900 mm^3^ tumors). Extracted tumors from a cohort of 3 mice per group were minced and the chunks were incubated at 37 °C and 5% CO_2_ in tumor cell growth media for 2 hours, and the resulting media was analyzed for cytokines (IFN-γ, IL-2, TNFα, TGFβ, and IL-1α) by ELISA according to the manufacturer’s instructions (Biolegend, CA).

### Vaccination and animal study

7-week-old female C57BL/6 mice (Jackson Laboratory, ME) were housed in a pathogen-free facility in the vivarium of Binghamton University. All animal study procedures were approved by the Institutional Animal Care and Use Committee (IACUC) at Binghamton University. All experiments and methods were performed in accordance with relevant guidelines and regulations. 5 × 10^5^ B16F10 cells were implanted on the left flank of 7-week-old female C57BL/6 mice to induce the tumor development. The tumors were measured using calipers every alternate day after tumor growth was observed. To prepare whole cell vaccines, CD47^−/−^ B16F10 cells (Referred to as CD47^−/−^ 3BD9) and CD47^+/+^ B16F10 cells (Referred to as B16 WT) were irradiated with 35 Gy gamma irradiation using a Cs source (University of Rochester Medical Center).

Mice (15 per group) were vaccinated with 5 × 10^5^ irradiated 3BD9 or B16 WT cells subcutaneously on the left flank and were then challenged with 5 × 10^5^ live B16F10 cells 7 days later.

Tumors and TDLNs at three different stages of tumor growth– small (200–300 mm^3^), medium (500–600 mm^3^), and large (800–900 mm^3^) – were collected from five mice per group after euthanasia by CO_2_ inhalation. For the mice that did not develop tumors, TDLNs alone were collected at day 90 post tumor challenge. Organs were enzymatically digested and prepared into single cell suspensions for immunostaining.

### Immunophenotyping

To determine the types of tumor infiltrating lymphocytes (TILs) and TDLN lymphocytes, single cell suspensions of the tumors and TDLNs were stained using two multicolor panels covering the APC compartment - macrophages (M_φ_), dendritic cells (DCs), myeloid derived suppressor cells (MDSCs), and monocytes; and the effector cell compartment - cytotoxic T cells (CTLs), helper T cells (T_H_ cells), memory cells, regulatory T cells (T-regs), natural killer (NK) cells, and activated effector cells. All pre-conjugated antibodies were purchased from Biolegend unless otherwise specified. Samples were run on LSR Fortessa flow cytometers (University of Rochester Medical Center Flow Core Facility) and analyzed using the FlowJo software v10 (TreeStar).

### Statistical analyses

All statistical analyses were performed on GraphPad Prism. The non-parametric Mantel-Cox test was used for survival and tumor-free mice data. For other correlative analyses either a one-way ANOVA or an unpaired *t* test was performed based on the number of groups being compared. The Shapiro-Wilk test was used to determine population distribution when necessary. 95% confidence interval was used in all analyses to accept or reject the null hypothesis.

## Supplementary information

Supplementary Information

## Data Availability

The raw or analyzed datasets for this study are available from the corresponding author upon reasonable request.
