# Supplementary material for: Inducing Tumor Suppressive Microenvironments through Genome Edited CD47−/− Syngeneic Cell Vaccination
Source: Sci Rep. 2019 Dec 27;9:20057. doi: 10.1038/s41598-019-56370-6 (PMC6934648; doi:10.1038/s41598-019-56370-6)
Supplement: Supplementary file 1 — Supplementary Information [file 41598_2019_56370_MOESM1_ESM.docx]

**Inducing Tumor Suppressive Microenvironments through Genome Edited CD47^-/-^ Syngeneic Cell Vaccination**

Subhadra Jayaraman Rukmini, Huanjing Bi, Puloma Sen, Benjamin Everhart, Sha Jin, and Kaiming Ye*

Department of Biomedical Engineering

Center of Biomanufacturing for Regenerative Medicine

Watson School of Engineering and Applied Science

Binghamton University, State University of New York (SUNY)

Binghamton, NY 13902-6000, USA

__________________

*Corresponding author: Kaiming Ye, Ph.D., Department of Biomedical Engineering, Center of Biomanufacturing for Regenerative Medicine, Watson School of Engineering and Applied Science, Binghamton University, State University of New York (SUNY), PO Box 6000, Binghamton, NY 13902. Phone: 607-777-5887; Fax: 607-777-5780; email: [kye@binghamton.edu](mailto:kye@binghamton.edu)

**SUPPLEMENTARY METHODS**

***CRISPR knockout***

***Plasmid cloning*** - The pSpCas9-BB-2A-GFP (PX458) (Addgene, MA) was used. The required gRNA sequence (in the form of a ligated double stranded DNA, Invitrogen, CA) was subcloned into the cloning site of the plasmid under a U6 promoter. The cloning site was flanked by restriction sites for BbsI enzyme (New England Biolabs, MA). *Gene deletion* - Two gRNAs were used, targeting Exons 1 and 2 of the *cd47* gene. The distance between the cut sites for these gRNAs was approximately 12.9 kb. Confirmation of knockout was done using a PCR primer set (MA-FP/MC-RP) (Eurofins Genomics, KY) flanking the cut sites. The deletion of the 12 kb fragment was visually analyzed by PCR (BioRad, CA) and DNA electrophoresis.

***Nucleofection of RNPs*** - For knocking out CD47 from GVAX cells (GM-CSF producing B16F10 cells), the ribonucleoprotein (RNP) method was used. gRNA (in the form of mRNA, Integrated DNA Technologies, IA)) was complexed with purified Cas9-3NLS protein *in vitro* (Integrated DNA Technologies, IA). The mixture was then electroporated into GVAX cells using the Neon Electroporation System (Invitrogen, CA). The cells were analyzed by flow cytometry (BD FACS Aria II) 6-9 days post electroporation. CD47^-/-^ cells were sorted for single cell colonies into 96-well plates using the BD FACS Aria II Cell Sorter. GVAX 1FC was chosen as the CD47^-/-^ clone for further studies.

***Organ preparation and immunophenotyping***

***Tumors*** - Tumors were harvested at the three stages as mentioned above, stored, and transported in media containing RPMI 1640 (Gibco), 2% FBS (Sigma), and Pen-Strep antibiotic (100 units Penicillin, 100 µg Streptomycin - Gibco) on ice. Tumors were then digested using 1 mg/mL collagenase and 10 mg/mL DNase (Sigma) at 37ᵒC for one 20-min cycle with intermittent high-speed vortexing, and another 20-min cycle with intermittent vigorous pipetting. Homogenized suspensions were filtered using a 70 µm filter and suspended in a FACS Buffer (1X PBS, 2% FBS, 2mM EDTA (Gibco), and 25mM HEPES (Gibco)) until use.

***Lymph nodes*** - TDLNs were harvested from mice along with the tumors and transported separately in media containing RPMI 1640 (Gibco), 2% FBS (Sigma), and Pen-Strep antibiotic (100 units Penicillin, 100 µg Streptomycin - Gibco) on ice. TDLNs were cut into small pieces using a sharp scalpel and 26 G needle (BD Biosciences). Fragments were placed in 2 mL warm Digest Buffer containing 1 mg/mL collagenase and 10 mg/mL DNase and digested at 37ᵒC for 30 min with intermittent high-speed vortexing. Fragments were filtered using a 70 µm filter and suspended in the FACS Buffer until use.

***Immunostaining*** - Approximately 2-4 million cells from tumors and 0.5-1 million cells from TDLNs were transferred to non-treated U-bottom 96-well plates (Costar). Two multicolor panels were used for tumors and TDLNs - *Panel T1* for tumors and *Panel SL1* for TDLNs (containing all antibodies for phenotyping the antigen presenting cell (APC) compartment, and tumor antigens), and *Panel P2* for both (containing an antibody cocktail for phenotyping the effector cell compartment). All the antibodies used in this experiment were titrated using mouse melanoma tumors and the dilutions were optimized for use in the multicolor panels. CD16/CD32 Fc Block (Biolegend, CA) was used to block immune cell receptors along with the antibody cocktails. Cells were stained for extracellular antigens in FACS Buffer for 30 min on ice, and then fixed and permeabilized using the Transcription Factor Buffer Set (BD Biosciences) for 30 min at 4ᵒC. The intracellular antigen staining was performed using the Perm/Wash Buffer from the set for 30 min at 4ᵒC. Stained cells were stored in the dark at 4ᵒC in the FACS Buffer until use. Multicolor compensation was performed by staining polymer beads from the AbC Antibody Compensation Kit (Invitrogen) with the appropriate amounts of all antibodies used in the staining panels.

**SUPPLEMENTARY FIGURES AND TABLES**

**Supplementary Table 1:** Guide RNA and PCR primer sequences designed for CD47 knockout and screening

| ID | Sequence | Location/Function |
| --- | --- | --- |
| CRISPR gRNA Sequences | | |
| Guide M1 | AACCGCCGCCGCGACAACGA | Exon 1, *cd47* gene, used for RNP electroporation, and in PX458 plasmid for gene deletion |
| Guide M3 | TGCTTTGCGCCTCCACATTA | Exon 2, *cd47* gene, used in PX458 plasmid for gene deletion |
| PCR Primers | | |
| MA-FP | AGCCAGAGGGAAGGAGTT | Forward primer, upstream of Guide M1 targeted *cd47* gene |
| MC-RP | CCACTTGCCCAAGAAGAG | Reverse primer, downstream of Guide M3 targeted *cd47* gene (with MA-FP, amplicon length: 12,986 bp in intact, and 473 bp in a deleted *cd47* gene) |

**Supplementary Table 2: Phenotypic characterization of immune cell subsets in the tumor microenvironment and the draining lymph nodes.** The table lists all the phenotypic subsets used in this project to determine immune cell function associated with tumor rejection and escape. Categorization of these subsets is based on their lineage, function, and specific cell surface markers.

| **Subset** | **Significance** | **Phenotype** | **Subset** | **Significance** | **Phenotype** |
| --- | --- | --- | --- | --- | --- |
| **Macrophage Subsets** | | | **Cytotoxic T Cell (CTL) Subsets - CD8^+^** | | |
| **Activated Macrophages** | Anti-tumorigenic; enhanced IL-2 production | CD11b^+^CD80^+^ | **Activated CTLs** | Primed tumor-specific CD8^+^ T cells | CD8^+^CD69^+^ |
| **M1-type Macrophages** | Anti-tumorigenic; efficient antigen presentation | CD11b^+^ CD80^+^ MHC-II^hi^ | **Activated/Proliferating CTLs** | Activated and proliferating CD8^+^ T cells; produce granzymes, perforins, IFN-γ | CD8^+^Ki67^+^ |
| **M2-type Macrophages** | Pro-tumorigenic; suppressed antigen presentation | CD11b^+^ CD206^+^ MHC-II^low^ | **Exhausted CTLs** | Deactivated phenotype of CD8^+^ T cells; incapable of cytotoxic killing | CD8^+^PD-1^+^ |
| **Suppressive Macrophages** | T cell deactivation | CD11b^+^ PD-L1^+^ | **Helper T Cell (T_H_) Subsets - CD4^+^** | | |
| **Tumor-Associated Macrophage (TAM) Subsets** | | | **Regulatory T cells (T-regs)** | Inhibit proliferation and activation of CTLs and T_H_ cells | CD4^+^CD25^+^FoxP3^+^ |
| **TAM-1** | Pro-tumorigenic; inefficient antigen presentation | CD11b^+^ Ly6C^int^ MHC-II^hi^ | **Activated/Proliferating T_H_ cells** | Activated and proliferating CD4^+^ T cells; produce IL-2, IFN-γ | CD4^+^Ki67^+^ |
| **TAM-2 (MHC-II^hi^ TAMs)** | Pro-tumorigenic; normoxic conditions | CD11b^+^ Ly6C^low^ MHC-II^hi^ | **Exhausted T_H_ cells** | Deactivated phenotype of CD4^+^ T cells; incapable of cytokine release | CD4^+^PD-1^+^ |
| **TAM-3 (MHC-II^low^ TAMs)** | Pro-tumorigenic; present in hypoxic conditions; suppressed antigen presentation | CD11b^+^ Ly6C^low^ MHC-II^low^ | **Natural Killer (NK) Cell Subsets - CD49b^+^** | | |
| **Dendritic Cell (DC) Subsets** | | | **Activated Natural Killer Cells** | Primed NK cells; produce granzymes; cytotoxic activity | CD49b^+^Ki67^+^ |
| **Activated DCs** | Anti-tumorigenic; enhanced IL-2 production; efficient antigen presentation | CD11c^+^ MHC-II^+^ CD80^+^ | **Ratios of Cell Types** | | |
| **Migratory DCs** | Present mostly in tumor-draining lymph nodes; signifies high DC activity | CD11c^hi^ MHC-II^+^ CD11b^+^ | **Ratio of T-regs to Ki67^+^ T cells** | High ratio signifies a more suppressive environment; no T cell proliferation | - CD4^+^CD25^+^FoxP3^+^ : CD4^+^ Ki67^+^ - CD4^+^CD25^+^Ki67^+^ : CD8^+^Ki67^+^ |
| **Regulatory DCs** | May be pro-tumorigenic | CD11c^+^ MHC-II^+^ CD11b^+^ |  |  |  |
| **Suppressive DCs** | T cell deactivation | CD11c^+^ PD-L1^+^ |  |  |  |
| **Myeloid-Derived Suppressor Cell (MDSC) Subsets** | | |  |  |  |
| **Poly-morphonuclear (PMN) MDSCs (granulocytic)** |  | CD11b^+^ Ly6C^low^ Ly6G^+^ |  |  |  |
| **Monocytic (Mo) MDSCs** |  | CD11b^+^ Ly6C^hi^ Ly6G^-^ |  |  |  |
| **Monocyte-derived (Mo-derived) MDSCs** |  | CD11b^hi^ Ly6G^-^ MHC-II^+^ |  |  |  |

**Supplementary Table 3:** Antibodies used for immunophenotyping lymphocyte populations in TME and TDLNs

| **No.** | **Marker** | **Antibody Clone** | **Fluorophore** | **Dilution** | **Concentration in Panel (µg/ml)** |
| --- | --- | --- | --- | --- | --- |
| **Panel T1 - Tumor Cells and Antigen Presenting Compartment - TME** | | | | | |
| 1 | **CD45** | 30-F11 | PerCP-Cy5.5 | 1:300 | 0.7 |
| 2 | **CD11b** | M1/70 | Alexa Fluor 488 | 1:200 | 1 |
| 3 | **Ly6C** | HK1.4 | Brilliant Violet 785 | 1:300 | 0.7 |
| 4 | **Ly6G** | 1A8 | PE | 1:200 | 1 |
| 5 | **CD11c** | N418 | Brilliant Violet 421 | 1:200 | 1 |
| 6 | **CD47** | miap301 | Alexa Fluor 647 | 1:100 | 2 |
| 7 | **PD-L1** | 10F.9G2 | Brilliant Violet 605 | 1:100 | 2 |
| 8 | **CD80** | 16-10A1 | Brilliant Violet 650 | 1:100 | 2 |
| 9 | **MHC-II** | M5/114.15.2 | APC/Fire750 | 1:200 | 1 |
| **Panel SL1 - Antigen Presenting Compartment - TDLN** | | | | | |
| 1 | **CD45** | 30-F11 | PerCP-Cy5.5 | 1:300 | 0.7 |
| 2 | **CD11b** | M1/70 | Alexa Fluor 488 | 1:200 | 1 |
| 3 | **Ly6C** | HK1.4 | Brilliant Violet 785 | 1:300 | 0.7 |
| 4 | **Ly6G** | 1A8 | PE | 1:200 | 1 |
| 5 | **CD11c** | N418 | Brilliant Violet 421 | 1:200 | 1 |
| 6 | **CD80** | 16-10A1 | Brilliant Violet 650 | 1:100 | 2 |
| 7 | **MHC-II** | M5/114.15.2 | APC/Fire750 | 1:200 | 1 |
| 8 | **CD206** | C068C2 | Alexa Fluor 647 | 1:100 | 2 |
| **Panel P2 - Effector Cell Compartment - TME and TDLN** | | | | | |
| 1 | CD45 | 30-F11 | PerCP-Cy5.5 | 1:300 | 0.7 |
| 2 | CD8a | 53-6.7 | Brilliant Violet 510 | 1:100 | 2 |
| 3 | CD69 | H1.2F3 | Brilliant Violet 650 | 1:100 | 2 |
| 4 | CD4 | GK1.5 | Brilliant Violet 785 | 1:200 | 1 |
| 5 | CD25 | 3C7 | APC | 1:100 | 2 |
| 6 | FoxP3 | FJK-16s | Alexa Fluor 488 | 1:100 | 2 |
| 7 | CD49b | CX5 | PE-CF594 | 1:100 | 2 |
| 8 | PD-1 | RMP1-14 | PE | 1:200 | 1 |
| 9 | Ki-67 | 16A8 | Brilliant Violet 421 | 1:200 | 1 |

**SUPPLEMENTARY FIGURES**

**Supplementary Fig. 1:**


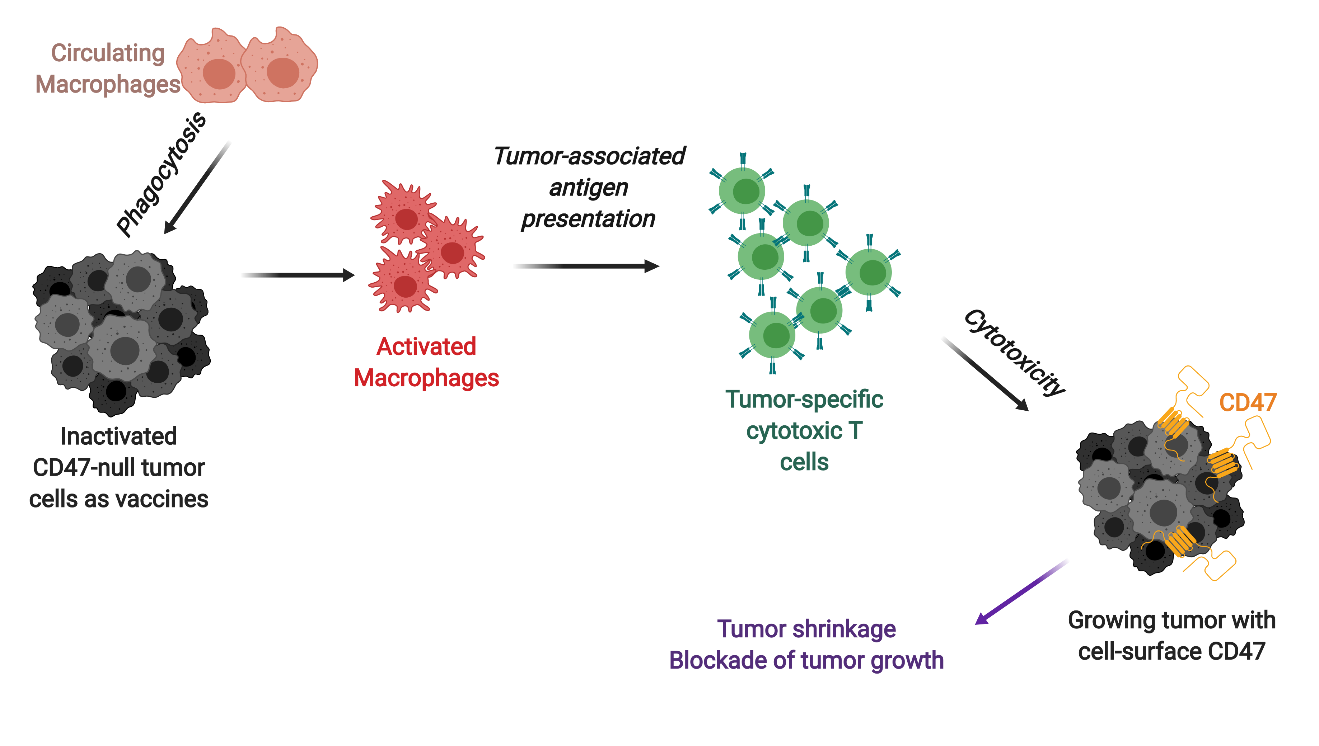


**Supplementary Fig. 1: The working mechanism of CD47-null whole-tumor-cell vaccines.** The diagram represents the proposed mechanism of action of our CD47-null whole-tumor-cell vaccines. When the immune system is presented with CD47-null inactivated syngeneic tumor cells, it is unmasked to the circulating macrophages, which then phagocytose the tumor cells and present tumor-specific antigens effectively to T cells. In the downstream immune response cascade, primed effector cytotoxic T cells infiltrate a syngeneic growing tumor, leading to tumor cell cytotoxicity and reduction of tumor growth. The presence of cell surface CD47 on the growing tumor does not affect the anti-tumor T cell response.

**Supplementary Figure 2:**


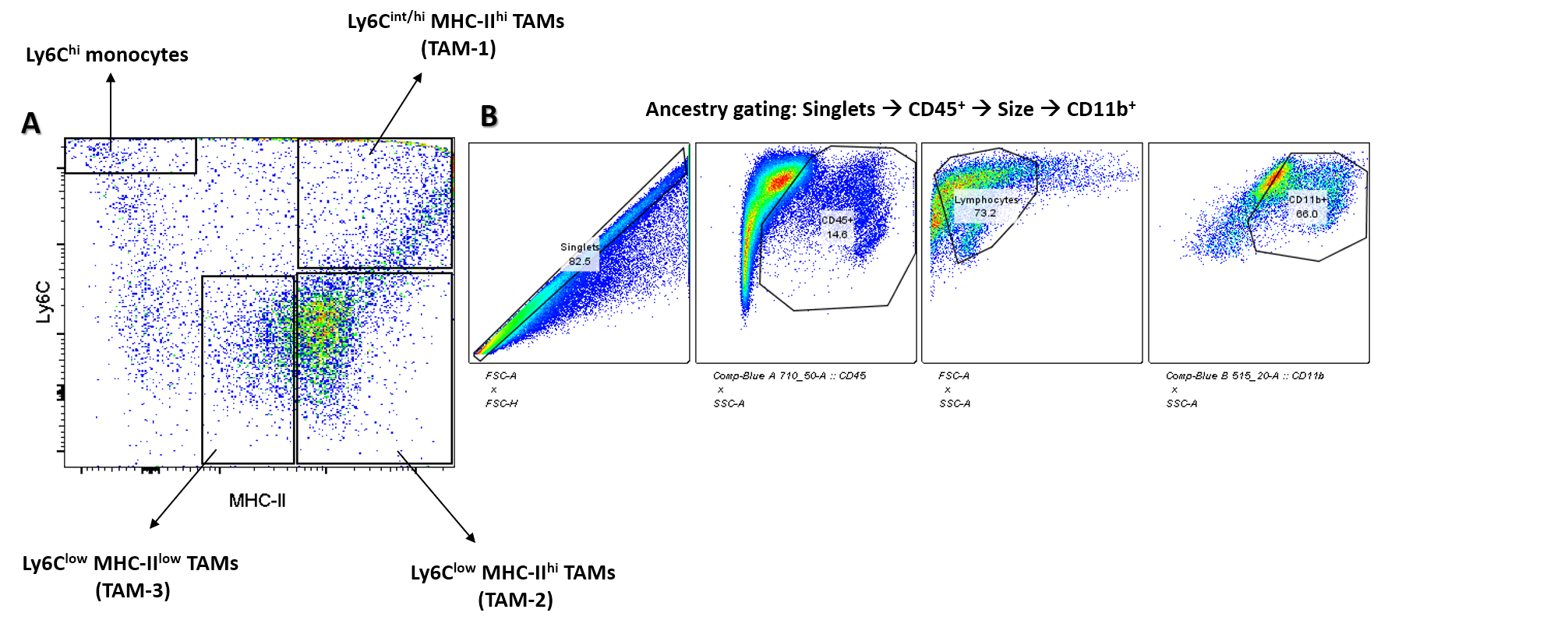


**Supplementary Fig. 2: Gating strategy for detecting tumor-associated macrophages (TAMs) in the TME.** (A) A pseudo-color scatter plot of MHC-II expression against Ly6C expression by tumor infiltrating lymphocytes. Gates were drawn according to expression levels of the markers based on FMO controls. The populations were categorized based on cells expressing various levels of each marker on their cell surface. (B) A panel of four scatter plots showing the ancestry of back-gating of the cell population to arrive at the graph in (A). Cells were first gated based on their forward scatter (FSC) to filter out doublets and clumps. They were then gated for the expression of the pan-lymphocyte marker CD45 on their cell surface. The next gating was based on cell size, and the last step before categorizing cells as TAMs, was gating for the expression of the macrophage marker CD11b.
